# Dengue Virus Nonstructural Protein 5 (NS5) Assembles into a Dimer with a Unique Methyltransferase and Polymerase Interface

**DOI:** 10.1371/journal.ppat.1005451

**Published:** 2016-02-19

**Authors:** Valerie J. Klema, Mengyi Ye, Aditya Hindupur, Tadahisa Teramoto, Keerthi Gottipati, Radhakrishnan Padmanabhan, Kyung H. Choi

**Affiliations:** 1 Department of Biochemistry and Molecular Biology, Sealy Center for Structural Biology and Molecular Biophysics, University of Texas Medical Branch at Galveston, Galveston, Texas, United States of America; 2 Department of Microbiology and Immunology, Georgetown University School of Medicine, Washington, D.C., United States of America; Institut Pasteur, FRANCE

## Abstract

Flavivirus nonstructural protein 5 (NS5) consists of methyltransferase (MTase) and RNA-dependent RNA polymerase (RdRp) domains, which catalyze 5’-RNA capping/methylation and RNA synthesis, respectively, during viral genome replication. Although the crystal structure of flavivirus NS5 is known, no data about the quaternary organization of the functional enzyme are available. We report the crystal structure of dengue virus full-length NS5, where eight molecules of NS5 are arranged as four independent dimers in the crystallographic asymmetric unit. The relative orientation of each monomer within the dimer, as well as the orientations of the MTase and RdRp domains within each monomer, is conserved, suggesting that these structural arrangements represent the biologically relevant conformation and assembly of this multi-functional enzyme. Essential interactions between MTase and RdRp domains are maintained in the NS5 dimer via inter-molecular interactions, providing evidence that flavivirus NS5 can adopt multiple conformations while preserving necessary interactions between the MTase and RdRp domains. Furthermore, many NS5 residues that reduce viral replication are located at either the inter-domain interface within a monomer or at the inter-molecular interface within the dimer. Hence the X-ray structure of NS5 presented here suggests that MTase and RdRp activities could be coordinated as a dimer during viral genome replication.

## Author Summary

Many plus-strand RNA viruses encode a viral RNA polymerase and capping enzymes to synthesize a 5’-capped RNA genome. However, how these two activities are coordinated during viral replication is not understood. In flaviviruses, polymerase and capping enzymes are encoded in a single multifunctional protein, where separate domains within the polypeptide are responsible for these activities; flavivirus NS5, composed of the polymerase and methyltransferase domains, carries out viral RNA synthesis, 5’-RNA capping, and RNA cap methylations. Previous NS5 monomer structures were unable to provide mechanistic insight into how the two domains communicate or the quaternary organization of the functional enzyme. We have determined the crystal structure of dengue virus NS5 and show that the NS5 dimer is likely the biological assembly of NS5, and RNA synthesis and RNA capping may be coordinated by the dimer. We found that essential interactions between the two NS5 domains can be maintained either within a monomer or via inter-molecular interactions within a dimer, and thus NS5 can adopt multiple conformations while preserving necessary interactions between the methyltransferase and polymerase domains. Using dengue virus, we additionally determined that such specific interaction between the two NS5 domains is the major determinant of viral replication.

## Introduction

*Flaviviridae* includes at least 70 mosquito- and tick-borne viral species, some of which act as the etiological agents of human diseases such as dengue virus (DENV), West Nile virus (WNV), Japanese encephalitis virus (JEV), and yellow fever virus (YFV). Dengue is the most prevalent mosquito-borne virus, with nearly 400 million annual cases worldwide [1]. Infection with one of four dengue virus serotypes (DENV1-4) can lead to febrile illness and flu-like symptoms, or can progress to the more severe dengue hemorrhagic fever or dengue shock syndrome. Development of the more severe hemorrhagic fever is more likely if recovery from infection by one dengue serotype is followed by subsequent infection by a second serotype [2]. There are currently no effective antiviral drugs for the treatment of flavivirus infections, and no vaccine is available for protection against severe diseases caused by any of the DENV serotypes or WNV [3].

The 10–11 kb positive (+) sense flaviviral genome consists of a single open reading frame (ORF) flanked by 5’ and 3’ untranslated regions (UTR). The 5’ terminal nucleotide A is modified by the addition of a type 1 cap (^N7Me^G5’-ppp-5’A_2’OMe_). The viral ORF is translated into a single polyprotein that is co- and post-translationally cleaved by host and viral proteases to produce ten proteins, including three structural proteins (capsid, pre-membrane, and envelope) and seven nonstructural (NS) proteins (NS1, NS2A, NS2B, NS3, NS4A, NS4B, and NS5) [4]. All NS proteins, along with the viral RNA and cellular factors, form a viral replication complex embedded within virus-induced membrane vesicles located at the rough endoplasmic reticulum of infected cells [5–8]. NS3 and NS5 are the key enzymes in the replication complex, as together they account for all catalytic activities required for genome replication and RNA capping at the 5’ UTR. NS3 consists of an N-terminal serine protease domain, which requires NS2B as a cofactor, and a C-terminal helicase domain possessing three distinct activities: RNA helicase, nucleoside triphosphatase, and 5’ RNA triphosphatase [4]. NS5, the largest NS protein at 103 kDa, consists of an N-terminal methyltransferase (MTase) domain possessing three activities necessary for cap synthesis (guanylyltransferase, guanine-N7-methyltransferase, and nucleoside-2’O-methyltransferase) and a C-terminal RNA-dependent RNA polymerase (RdRp) domain that carries out *de novo* RNA synthesis [9–14]. Replication of the (+) strand RNA genome is an asymmetric and semi-conservative process wherein the antigenome is present only as a double-stranded (ds) RNA replication intermediate [15]. After (+) strand RNA synthesis by the NS5 RdRp, the cap structure is added to the 5’ end of the genome by four enzymatic reactions catalyzed by NS3 and NS5 [16]. First, the 5’ γ-phosphate of the (+) RNA is cleaved by NS3 5’-RNA triphosphatase activity to produce a diphosphate-terminated RNA (pp-5’A). Next, the NS5 MTase catalyzes the transfer of a GMP moiety from GTP to the RNA (G5’-ppp-5’A). Finally, NS5 MTase catalyzes sequential guanine-N7- and nucleoside-2’O-methylations using *S*-adenosyl-L-methionine (SAM) as a methyl donor and produces a type 1 cap structure (^N7Me^G5’-ppp-5’A_2’OMe_) [16,17].

The physical linkage of two distinct catalytic domains within NS5 suggests that RNA synthesis and genome capping activities may be coupled during viral replication [18]. Communication between the MTase and RdRp domains has indeed been observed in several flaviviruses via reverse genetic, biochemical, and structural experiments [19–24]. Here we present the crystal structure of full-length DENV3 NS5. A total of eight copies of full-length NS5 are arranged as four dimers within the crystallographic asymmetric unit (ASU). All eight molecules have essentially the same relative orientation of the MTase and RdRp domains, and this arrangement differs from that seen in JEV NS5 [25]. In the structure presented here, the linker region between the MTase and RdRp domains is fully resolved, and comparison with the JEV NS5 structure suggests that this region acts as a hinge that allows NS5 to adopt multiple conformations. Furthermore, many residues that reduce viral replication are located at the inter-domain and inter-molecular interfaces, and thus the domain-domain interactions within a monomer as well as monomer-monomer interactions within a dimer likely play a role during viral genome replication. The DENV NS5 structure will aid the development of structure-based inhibitors that would interfere with inter-domain or inter-molecular interactions.

## Results

### DENV NS5 arranges as a dimer with identical inter-domain and inter-molecular interfaces

**Fig 1:**
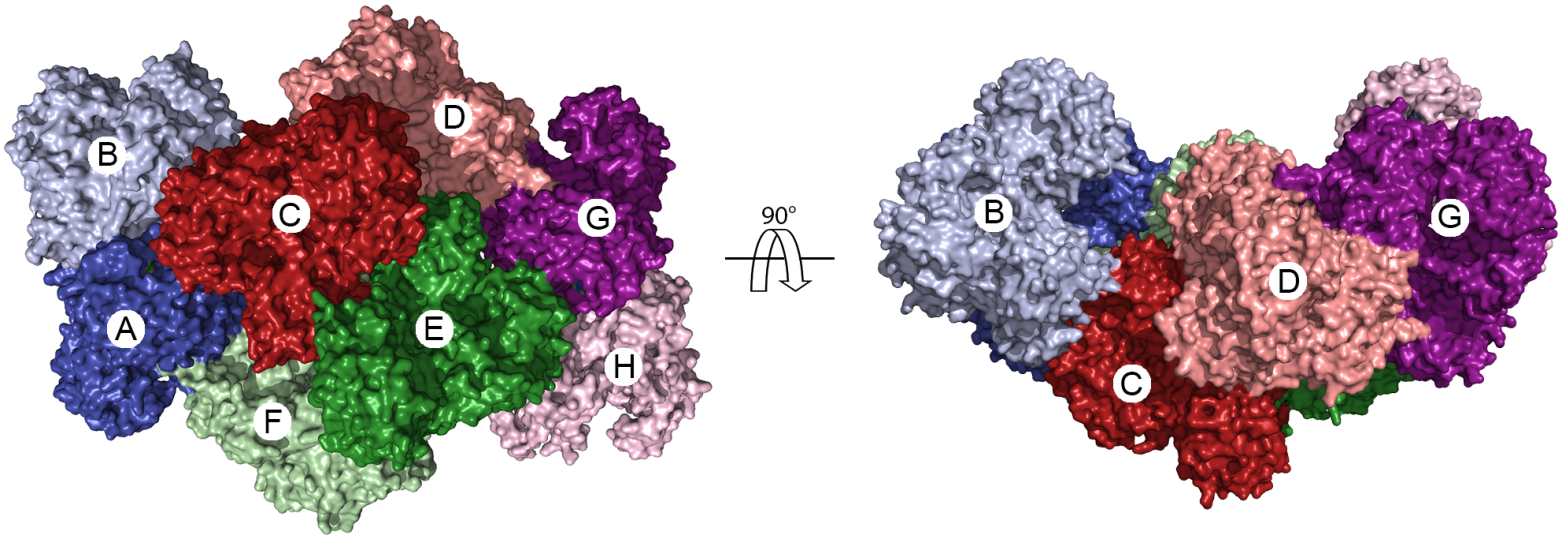
Assembly of DENV NS5. The crystallographic asymmetric unit of DENV NS5 contains eight monomers that assemble into four homodimers. Monomers are shown as molecular surfaces, and dimers are indicated using two shades of one color. The non-crystallographic 2-fold symmetry axis is indicated.

The crystallographic ASU contains eight copies of full-length DENV3 NS5, in which four sets of dimers (AB, CD, EF, and GH) are arranged in a saddle-like shape (Fig 1). The AB and CD dimers are related to the GH and EF dimers, respectively, by a 2-fold non-crystallographic symmetry (NCS) axis. Superimposition of the eight NS5 monomers and four NS5 dimers within the ASU yields rmsd values ranging from 0.4 to 1.9 Å, and from 0.8 to 2.3 Å, respectively, indicating that all copies of NS5 are nearly superimposable with one another. Thus the relative arrangement of MTase and RdRp domains within a monomer and the arrangement of monomers within a dimer are conserved in all eight molecules. These eight molecules within the ASU are subject to entirely different packing forces, hence the observed arrangement is not an artifact of crystallization (S1 Fig).

### The RdRp domain shows newly resolved structural features (motif G and the C-terminus)

**Fig 2:**
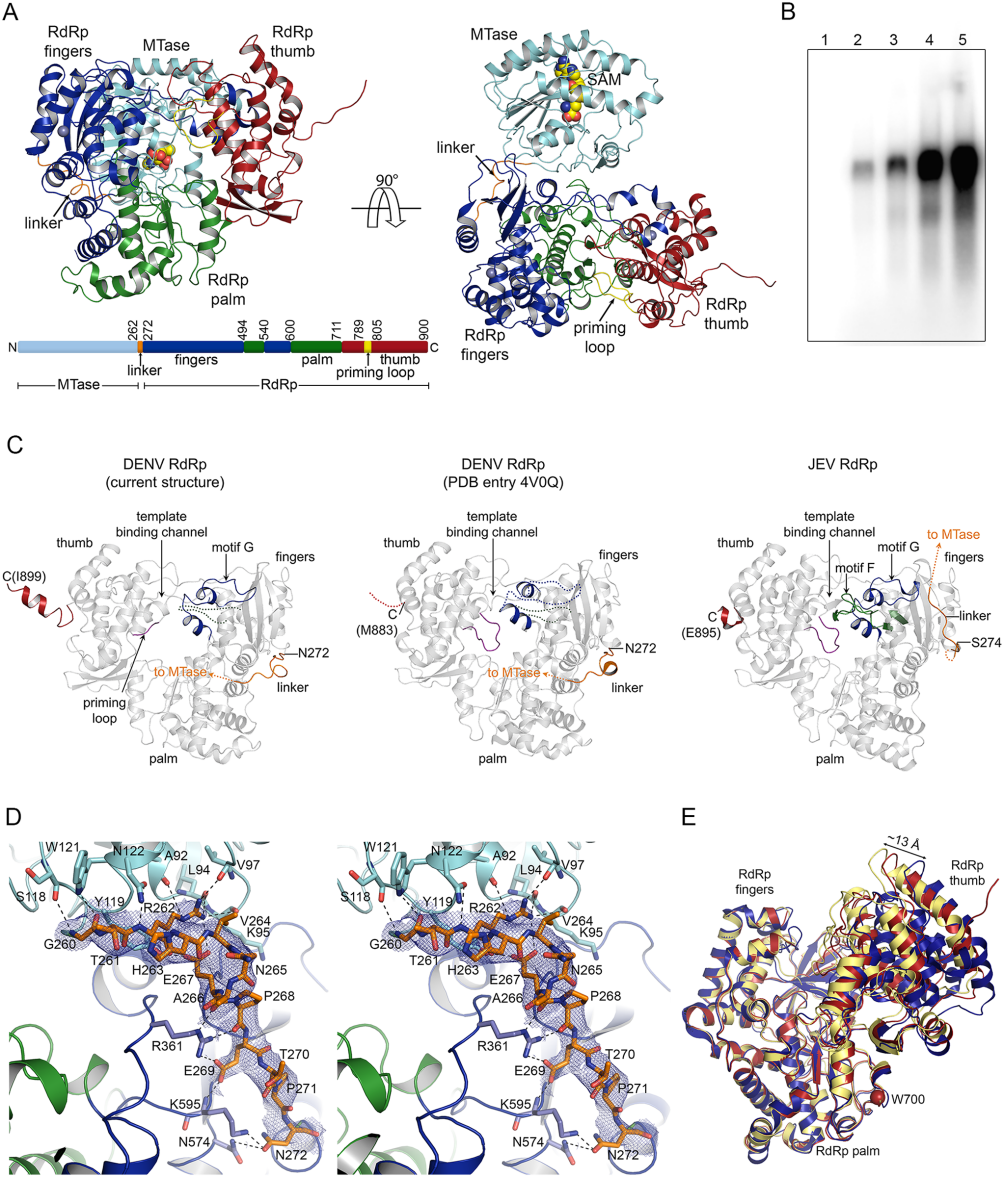
DENV NS5 monomer. **(A)** Overall fold of the DENV NS5 monomer. The ribbon diagram of the NS5 monomer is shown looking through the canonical right hand configuration (left) and through the top of the RdRp (right). The MTase domain is shown in cyan, and the RdRp domain is colored by region (thumb, red; palm, green; fingers, blue; domain linker, orange; priming loop, yellow). SAH is shown as a space-filling model and colored by atom type. Two zinc ions are shown as gray spheres. Schematic of the NS5 domains is shown below. **(B)** Polymerase activities of NS5 proteins. Polymerase activities of NS5 proteins were measured using a subgenomic RNA as a template, as previously described [27]. Lanes: 1, no polymerase; 2, NS5 RdRp domain; 3, wild-type NS5; 4, NS5-Δ2; 5, NS5-Δ6. Please see Materials and Methods for specific deletions. (C) Comparison of RdRp domains of DENV and JEV NS5. The RdRp domains of the two DENV (the current structure and PDB code 4V0Q) and JEV (PDB code 4K6M) are compared. The previously disordered regions including motif F (green), motif G (blue), linker (orange), and the C-terminus (red) are labeled. Since none of the DENV NS5 monomers included both motif G and the C-terminal helix, we created a model of the monomer by grafting motif G from monomer C onto monomer G, which included the C-terminal helix; the resulting model thus has both motif G and the C-terminus. Motif F in DENV NS5 is disordered and shown as a dotted line. The priming loop is shown in purple. **(D)** Stereoview of the linker between the MTase and RdRp domains. The residues 260–272 are shown in orange, and the final 2*F*_*o*_-*F*_*c*_ omit map for the linker region is shown as a blue mesh contoured at 1.0 σ. MTase and RdRp residues that interact with the linker are colored as in (A). Hydrogen bonds are indicated by dashed lines. **(E)** Movement of the thumb subdomain in flavivirus NS5. The RdRp domain of DENV NS5 (chain B, blue) was aligned with the JEV RdRp domain (orange) by fingers and palm subdomains (rmsd = 0.56 Å for 333 Cα atoms). The thumb subdomain of DENV NS5 is rotated by 7° around W700 (hinge residue, blue sphere) compared to the JEV thumb subdomain.

Individual MTase and RdRp domains in full-length NS5 have conserved protein folds. The MTase (residues 1–262) adopts the SAM-dependent methyltransferase fold composed of four helices surrounding a central 7-stranded β-sheet (Fig 2A), similar to previously determined flavivirus MTase structures [13]. The active site, containing a catalytic K61-D146-K180-E216 (KDKE) motif, is positioned in the center of the β-sheet. The MTase core fold is surrounded by N- and C-terminal extensions that interact with each other. Although no SAM or *S*-adenosyl-L-homocysteine (SAH, a byproduct of the methyltransferase reaction) were added during purification or crystallization, SAH is clearly visible in the electron density in the active site (S2 Fig). The C-terminal RdRp (residues 273–900) adopts the canonical right hand polymerase fold, consisting of fingers, palm, and thumb subdomains (Fig 2A). The palm subdomain contains a catalytic G662-D663-D664 (GDD) metal-binding motif. Flavivirus RdRps initiate RNA synthesis *de novo*, without the need of a primer. The priming loop in the thumb subdomain is proposed to stabilize the *de novo* initiation complex and occlude access to the template-binding channel [26]. During elongation, however, the priming loop is proposed to open to allow exit of dsRNA products, requiring significant movement. The NS5 construct used to solve the crystal structure (NS5-Δ6) contained a six-residue deletion (^795^WSIHAH^800^) in this priming loop, which was rationally designed to eliminate the need for this movement. This protein has higher polymerase activity than the wild-type (WT) protein when a subgenomic RNA is used as a template [27] (Fig 2B). Thus the structure represents a biologically relevant form of the polymerase. The shortened priming loop of the protein follows continuous density in the electron density map, suggesting that the deletion did not significantly affect the protein fold.

**Fig 3:**
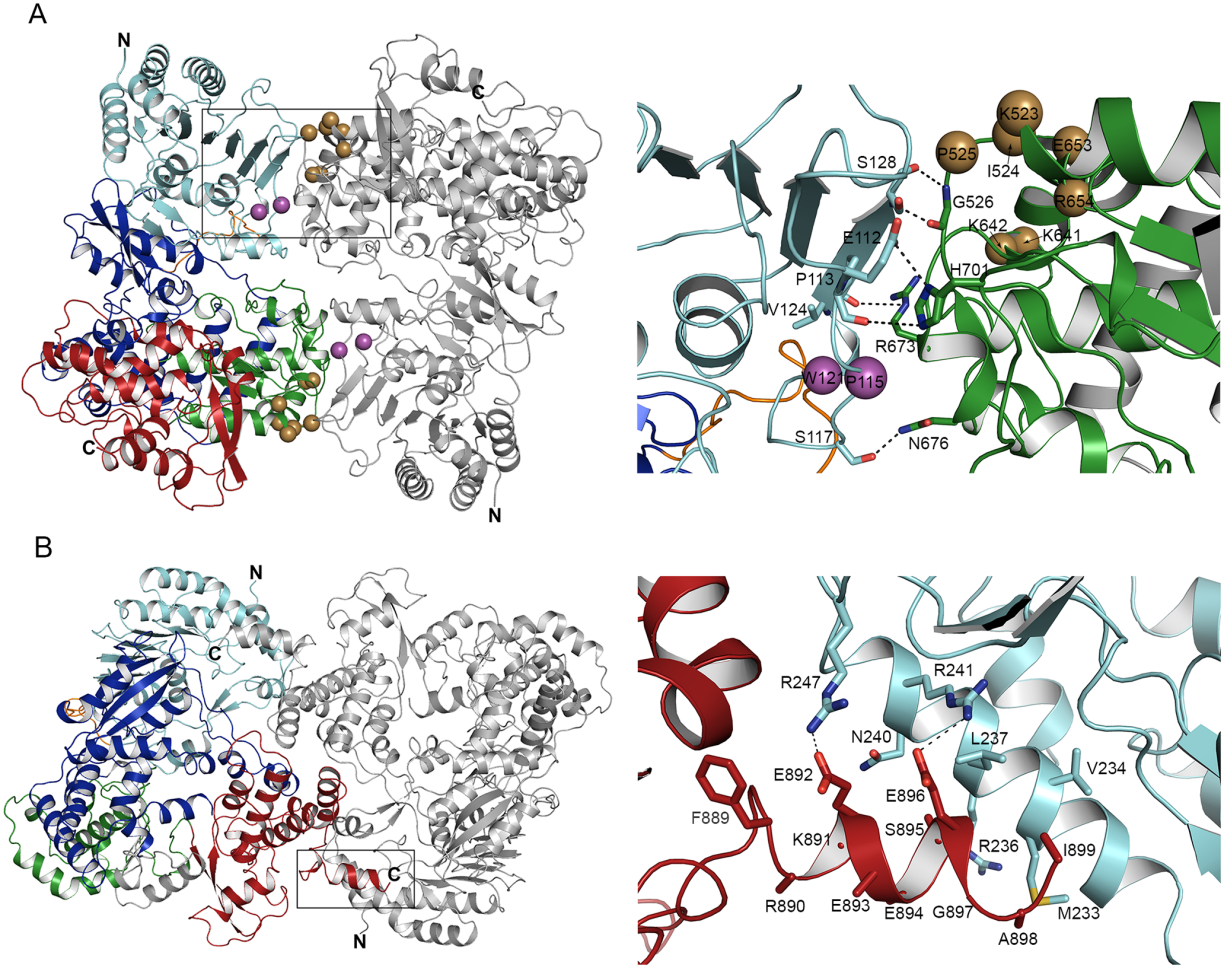
NS5 dimer interactions. **(A)** Type I dimer. All eight NS5 monomers are involved in type I dimer interaction (AB, CD, EF, and GH). One monomer is colored as in Fig 2A and the other is in gray. Residues of potential physiological significance are shown as magenta (MTase) or gold (RdRp) spheres, and labeled with corresponding one-letter residue codes. The close-up view of the type I dimer interface is shown as sticks (right), and hydrogen bonds are indicated by dashed lines. **(B)** Type II dimer. The type II dimers are observed only between monomers A and F and between D and G. The close-up view of the C-terminal residues involved in the dimer interface is shown as sticks (right).

Three areas of the DENV RdRp had been unresolved in the previous crystal structures [28–30]. While this manuscript was in preparation, a DENV NS5 structure was published [31], and thus was included in comparison. First, the region containing motif G (residues 408–417) in the fingers subdomain is disordered. Motif G is proposed to form a part of the template-binding channel and regulate RNA template binding and translocation [25]. Three monomers (B, C, and E) out of the eight copies in the DENV NS5 ASU have connected density for a long loop near the entrance to the template-binding channel (Fig 2C). This is the first time that motif G has been fully resolved in the DENV RdRp structures. The conformation of the loop is similar to that observed in JEV NS5 [25] (Fig 2C). Second, the fingertip region formed by residues 454 to 469 (motif F) is disordered in all eight copies. Motif F is well ordered in JEV NS5 and forms part of the template-binding channel near the MTase-RdRp interface (Fig 2C). Unexpectedly, when JEV and DENV RdRp domains are overlaid, motif F in JEV NS5 sterically clashes with the MTase in the DENV NS5 structure due to differences in the MTase and RdRp interface (see below). This suggests that motif F adopts several conformations depending on the interactions between MTase and RdRp domains. Finally, the C-termini (residues 884–899) of four monomers (A, D, F and G) were clearly resolved for the first time. The C-terminus forms an α-helix and interacts with the MTase of a neighboring NS5 (Figs 2C and 3B). Involvement of the C-terminus is further discussed in the intermolecular interactions below.

When the eight RdRp domains are overlaid, the biggest difference is the orientation of the thumb subdomain relative to the palm and fingers subdomains (Fig 2E). For example, the thumb subdomain in monomer B is rotated ~5° around a hinge point at residues G699 and W700 relative to the other monomers. This results in a 6 Å movement of an α-helix in the thumb, as calculated by the program DynDom [32]. When the RdRp domains were further compared to other flavivirus RdRp structures, movement of the thumb subdomain relative to the palm and fingers subdomains ranges up to 13 Å (Fig 2E). Such flexibility of the thumb subdomain could control the size of the template-binding channel to accommodate either an ssRNA template or a dsRNA product during RNA synthesis.

### DENV NS5 uses a novel MTase and RdRp interface

The unusually large number of molecules in the crystallographic ASU allows us to examine interactions of NS5 domains without the influence of crystal contacts. Although the individual NS5 molecules are not restricted by crystal lattice contacts, all eight copies of NS5 have the same relative orientation of the MTase and RdRp domains. Looking at the canonical right hand orientation of the RdRp, the MTase is located behind the RdRp near the template entry channel, opposite the priming loop (Fig 2A). In this arrangement of two domains, the active site of the MTase and the dsRNA exit of the RdRp face in opposite directions, exposed to solvent. The MTase interacts mainly with the fingers subdomain of the RdRp, and buries ~1000 Å^2^ of surface area as calculated by the program PISA [33]. Two areas of NS5 contribute to the inter-domain interface—the linker between the MTase and RdRp domains, and the interface involving two helices on the periphery of the MTase core and the backside of the fingers subdomain (Figs 2A and S3).

The MTase and RdRp domains are connected by a 10-residue linker (residues 263–272). The linker has very low sequence identity among flavivirus NS5 proteins (one identical residue out of 10 residues), and was structurally unresolved in both the crystal structures of individual MTase and RdRp domains, as well as in the full-length JEV NS5 structure [25,30]. Hence the linker was predicted to be flexible. In the current structure, the linker is well ordered, and has complete density connecting the MTase and RdRp domains (Fig 2D). The linker is in an extended conformation and makes extensive interactions with each domain. R262 and E267 form hydrogen bonds with the MTase core (A92, L94, K95, V97, N112, and Y119) (Fig 2D). Residues P268, E269, and N272 interact primarily with the fingers subdomain of the RdRp (R361, K595, and N574) (Fig 2D). The average temperature factor for the linker ranges from 80 to 127 Å^2^ amongst the eight monomers, and is similar to the temperature factor of the neighboring residues in each monomer. The interactions between the linker and NS5 domains support previous observations that incorporation of the linker (residues 263–272) into the DENV1-4 RdRp domains improves thermal stability compared to the RdRp domain alone [30].

Adjacent to the linker region, there are additional molecular contacts at the MTase and RdRp interface. These include a cation-pi interaction between R68 and F348, and salt bridges between E67 and R352, and between E252 and R352 (S3 Fig). Hydrogen bonding interactions are also observed between Q63 and R352, G93 and K300, and D256 and E356. Most of the residues involved in the domain-domain interactions are conserved, and thus the MTase and RdRp interactions are likely preserved in DENV NS5 from all four serotypes. Several residues located in the MTase and RdRp domain interface have previously been mutated and shown to reduce viral replication. For example, virus containing the double mutation K356A/E357A in DENV4 NS5 (corresponding to K355/E356 in DENV3 NS5) reduced viral replication 100-fold relative to the WT virus [22,34]. Similarly, no virus was detected after transfection of DENV2 RNA transcripts containing R353A/K358A (R352/K357 in DENV3) or E357A/K358A/D360A (E356/K357/D359 in DENV3) in NS5 [34]. A F349D substitution in DENV2 NS5 (F348 in DENV3 NS5) also severely impaired viral replication [22]. Thus the MTase and RdRp interactions shown in the crystal structure are likely important for viral replication.

Flavivirus NS5 is proposed to adopt multiple conformations from compact to more extended forms based on small-angle X-ray scattering (SAXS) [35]. Crystal structures of JEV and recently solved DENV NS5 (PDB entry 4V0Q) indeed show different arrangements of MTase and RdRp domains along with different linker conformations [25,31]. It was thus interesting that the arrangement of MTase and RdRp domains in our DENV NS5 structure was similar to those in the 4V0Q structure. Despite the different crystallization conditions (1.0 M succinic acid and 1% monomethylether 2000 in our condition vs. 0.2 M calcium acetate and 10–20% PEG 8000 in the 4V0Q structure) and symmetry of crystals (P3_2_21 with 8 molecules in ASU vs. P2_1_2_1_2 with one molecule in ASU), the two DENV NS5 structures can be superimposed with rmsd values of 1.0–2.5 Å for each of the eight NS5 monomers. The 10-residue linkers, including a short 3_10_ helix, are also similar and they can be superimposed with rmsd values of 0.4–0.6 Å (Fig 2C). We next compared the NS5 crystal structure to the SAXS profile, which showed an average radius of gyration (R_G_) and maximum dimension (D_max_) of 34 and 125 Å, respectively. The R_G_ and D_max_ of both DENV NS5 structures are calculated to be 31 and 98 Å, respectively, using the program CRYSOL [36]. Thus, the crystal structure would correspond to a compact form of NS5 [35]. Since both structures have the same domain arrangements within the monomer despite the protein’s ability to adopt multiple forms in solution, the compact form of NS5 may be a preferred structure at high protein concentration.

### NS5 assembles into unique dimers

The eight molecules of NS5 in the crystallographic ASU consist of four dimers (AB, CD, EF, and GH in Fig 1), in which the relative arrangement of both monomers is fixed (type I dimer). The four dimers can be superimposed with rmsd values ranging from 0.8 Å (between AB and GH dimers) to 2.3 Å (between EF and GH dimers). The periphery of the core MTase domain in one NS5 molecule interacts with the base of the palm subdomain in the second NS5 molecule, and buries 1100–1200 Å^2^ of surface area between monomers in each pair (Fig 3A). This extensive intermolecular dimer interaction even includes two hydrogen bonds between main chain atoms of S128 in the MTase of one monomer and G526 in the RdRp of the neighboring monomer as in a short β-sheet (Fig 3A). MTase residues E112, S117, and V124 in one monomer interact with RdRp residues H701, N676, and R673 in the neighboring monomer, respectively. P113 forms an additional hydrogen bond with H701. The type I dimer is also observed in the recently solved DENV NS5 structure (PDB code 4V0Q) [31]. Although the NS5 structure was reported to be a monomer, i.e., one molecule in the ASU, the structure shows a crystallographic dimer that is nearly identical to our type I dimer. The crystallographic dimer can be superimposed with the type I dimer with an rmsd of 2.0 to 3.1 Å (for 1599–1671 Cα carbons). As mentioned previously, crystallization conditions and the symmetry of the crystals are significantly different between the two crystal forms. Crystal contact areas of the five dimers (four dimers in the current structure and the crystallographic 4V0Q dimer) were determined using the program CONTACT with a 4 Å cutoff. The crystal contacts were also quite different for each of the five dimers (S1 Fig). Thus, assembly of DENV NS5 into the type I dimer is not an artifact of crystallization.

Because of the arrangement of dimers within the 8 copies of NS5 in the ASU, there is a second type of dimer interaction (type II dimer) only observed between monomers A and F, and between D and G (Fig 3B). In the type II dimer, the tip of the thumb subdomain of one NS5 molecule (where the fingertips and the thumb domain meet) interacts with the same area in the neighboring molecule. This interface includes mostly hydrophobic interactions and buries ~1600 Å^2^ of surface area. Interestingly, only the four NS5 monomers in type II dimers (A, D, F, and G) have the extended density for the C-terminus (residues 884–899), contributing to the highly buried surface area. The C-terminus of each of the four monomers forms an α-helix and is located near the C-terminal α-helix (residues 228–243) in the neighboring MTase (Figs 2C and 3B). The C-terminus of flavivirus NS5 is thought to be flexible because the amino acid sequences are less conserved (< 25% identity), and it has not been observed in any crystal structures. The current structure indicates that the C-terminus of NS5 can be ordered and mediate interactions with other NS5 molecules.

The two types of NS5 dimer interactions with significant interface areas (>1,000 Å^2^) suggest that NS5 may function as a dimer in the viral replication complex. Although recombinant NS5 is a monomer in solution [35,37], cellular interactions between NS5 molecules have been shown using pull-down and fluorescence resonance energy transfer (FRET) assays [19,38]. For instance, the WNV RdRp domain pulls down the MTase domain and full-length NS5, indicating that inter-domain and inter-molecular interactions exist for NS5 in cells [19]. FRET assays also show that NS5 homo-oligomerizes via its RdRp domain [38]. Thus, if multiple copies of NS5 are present in the replication complex, a specific dimer of NS5, such as one shown in the crystal structure, may be formed preferentially. We thus investigated whether any of the NS5 residues located at one of the dimer interfaces have been shown to be important for viral replication. Indeed, several mutations in or near the type I dimer interface severely reduced viral replication. Individual mutations of P113D and W121D in DENV2 NS5 almost completely disrupted viral replication [22,23]. Both P113 and W121 are conserved in all DENV NS5 sequences, and interact with H701 and G260 at the type I dimer interface, respectively (Fig 3A). Several paired charge-to-alanine mutations in DENV4 NS5 led to viral attenuation in mice [34]. A double mutant K524A/K525A or K525A/D526A in DENV4 NS5 (K523/I524/P525 in DENV3) reduced viral replication by ~100 fold [34]. Other pairs such as E642A/R643A and D654A/R655A in DENV4, corresponding to K641/K642 and E653/R654 located near the dimer interface in DENV3 NS5, reduced viral replication by ~10,000 fold (Fig 3A). Thus the intermolecular interactions observed in the NS5 type I dimer seem to be important for viral replication. None of the residues in the type II dimer interface have been included in published mutagenesis studies, and thus it is not clear whether the type II dimer also plays a role in viral replication.

### Flavivirus NS5 exhibits two different arrangements of MTase and RdRp domains

**Fig 4:**
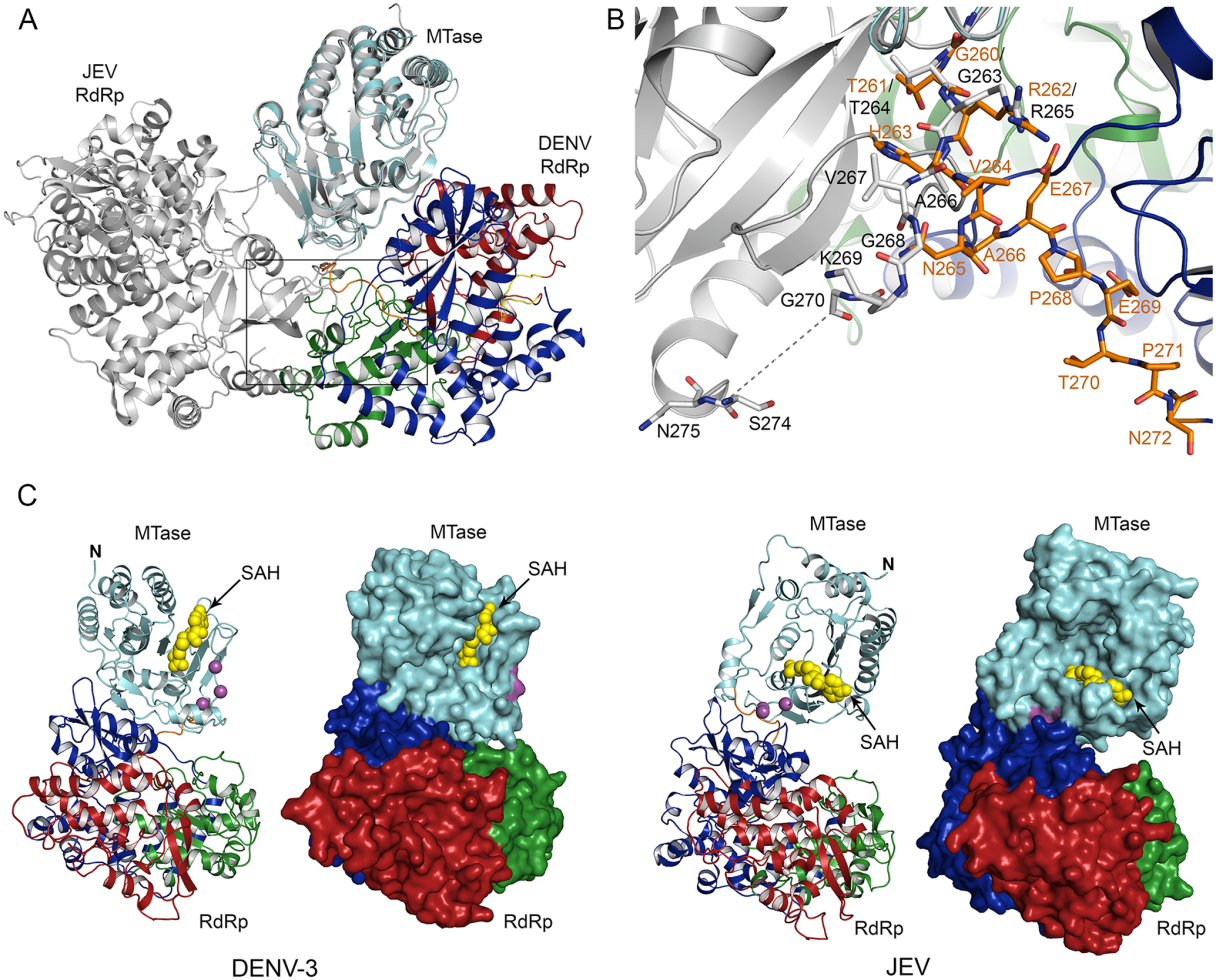
Comparison of DENV and JEV NS5 structures. **(A)** Superposition of DENV and JEV NS5. DENV and JEV NS5 (PDB code 4K6M) are aligned by the MTase domains only (rmsd of 0.99 Å for 253 Cα atoms). The DENV NS5 structure is colored as in Fig 2A, and the JEV NS5 is in gray. With the MTase domains superimposed, the RdRp domains of DENV and JEV NS5 are related by a rotation of 102° and a ~5Å translation along the linker region (residues 262–272). A close-up view of the boxed area is shown in (B). **(B)** The linker regions in DENV and JEV NS5. Residues comprising the linker regions of DENV and JEV NS5 are shown in orange and gray, respectively. Both structures use the ^260^GTR^262^ pivot, following which the two structures diverge. The dotted line indicates the break in the JEV NS5 linker. **(C)** Comparison of DENV and JEV NS5 structures. To emphasize the relative orientations of the MTase and RdRp domains, SAH molecules that occupy an identical binding site in both structures are shown in yellow. P113, P115 (or L115 in JEV NS5), and W121 in the MTase are implicated in viral replication and shown in magenta spheres in ribbon diagrams or magenta surfaces in surface representations. These residues in DENV NS5 are located in the dimer interface, while those in JEV NS5 are located in the domain interface.

The structure of DENV NS5 was compared to the JEV NS5 structure [25]. As in the DENV NS5 structure, the JEV MTase is positioned behind the RdRp when looking at the canonical right hand orientation of RdRp, and interacts with the fingers subdomain (Fig 4). However, the relative orientations of MTase and RdRp in DENV and JEV NS5 differ significantly. With the MTase of DENV and JEV NS5 superimposed (rmsd = 0.99 Å for 253 Cα atoms), the RdRp domains are related by a rotation of 102° and a ~5Å translation as calculated by DynDom [32] (Fig 4A). This twisting motion would require significant bending of residues 262–272 (the linker), using residues 260–262 (^260^GTR^262^) as the pivot (Fig 4B). The ‘GTR’ sequence is conserved in flavivirus NS5, and individual substitutions of G261A, T262V, and R263L in DENV2 and JEV NS5 greatly impair viral replication and virus production [22]. Thus, flavivirus NS5 likely uses the GTR pivot to sample different conformations, repositioning the MTase and RdRp domains during viral replication.

Due to the different arrangements of MTase and RdRp domains, DENV and JEV NS5 have different domain interfaces (Fig 4C). The domain interface in JEV NS5 buries ~860 Å^2^ surface area and centers around a hydrophobic core comprised of six residues: P113, L115, and W121 in the MTase domain, and F351, F467, and P585 in the RdRp domain. When individual mutations of the six residues were introduced into infectious JEV or replicon systems, virus replication was significantly impaired, while their polymerase activities were largely unaffected [22,23]. We mapped the corresponding residues on the DENV NS5 structure to determine whether they are involved in domain-domain interactions. The conserved RdRp residues F348 and P584 are found in the MTase and RdRp domain interface in the DENV NS5 monomer, similar to those involved in the intra-molecular interactions in the JEV NS5 monomer. F464 is disordered in the DENV NS5 structure. However, MTase residues P113, P115 (corresponding to L115 in JEV NS5) and W121 are not involved in domain-domain interactions in the DENV NS5 monomer (Fig 4C). Instead, the loop containing these MTase residues is part of the monomer-monomer interface in the NS5 type I dimer (Figs 3A and 4C). Thus, the same MTase region mediates intra- and inter-molecular interactions with the RdRp domain in JEV and DENV NS5, respectively. This surprising discovery again suggests that the inter-molecular interactions in the DENV NS5 dimer (type I) are likely important for viral replication.

### Serotype-specific interactions exist in the inter-domain interface and on the protein surface

**Fig 5:**
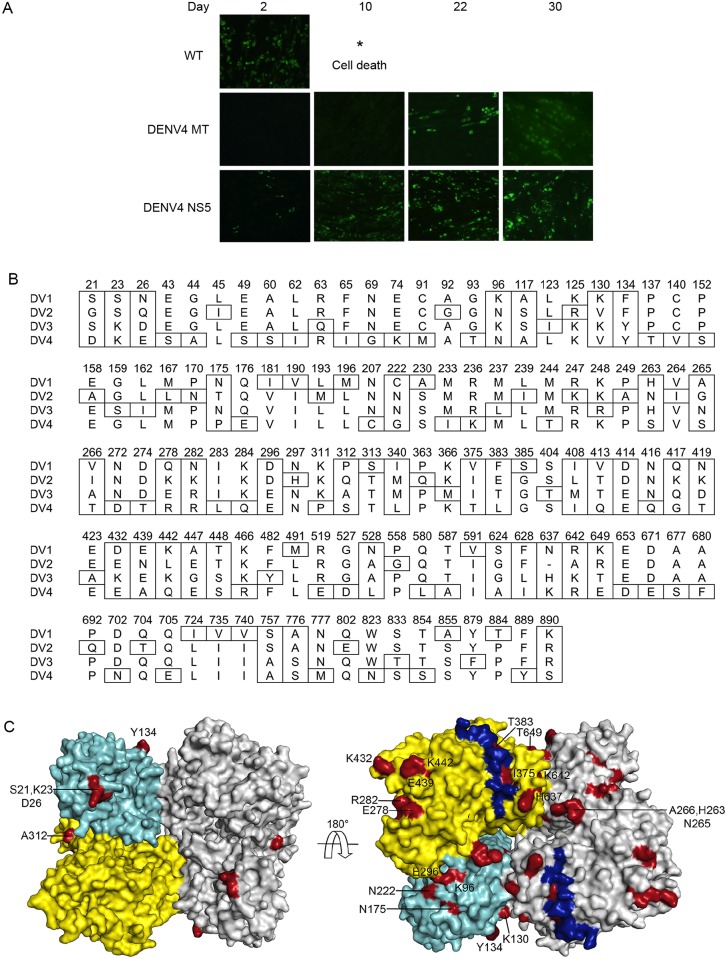
Serotype–specific interactions in DENV NS5. (A) Immunofluorescence assay. DENV2 RNA encoding the wild-type (DENV2) NS5, a chimera NS5 containing DENV4 MTase, or DENV4 NS5 were transfected into BHK-21 cells, and viral replication was visualized by immunofluorescence assay using anti NS1 antibodies. (B) Serotype-specific NS5 residues. Serotype-specific residues (boxed) are conserved within one DENV serotype, but not conserved across serotypes. Please see Methods section for details of sequence alignment and determination of serotype-specific residues. (C) Serotype-specific residues for at least two serotypes of DENV NS5 are mapped on the 3-D structure of the DENV3 NS5 dimer. One monomer is colored in cyan (MTase domain) and yellow (RdRp domain), and the other in gray. Serotype-specific residues are colored in red and labeled. The NLS (residues 369–406) is colored in blue.

NS5 is the most conserved protein in flavivirus, and sequence identities among DENV1-4 NS5 range from 74 (DENV2 vs. DENV4) to 84% (DENV1 vs. DENV3). Despite the high sequence identity, chimeric NS5, wherein the MTase and RdRp from different DENV serotypes or from DENV and WNV (66% sequence identity) are combined in a single polypeptide, is not capable of carrying out replication [39,40]. We have previously shown that a DENV2 infectious RNA containing an NS5 chimera with DENV2 MTase (residues 1–270) replaced by the DENV4 MTase was severely impaired and unable to accumulate viral RNA and virus particles [39]. Repeated passages of the chimeric RNA-transfected cells yielded viruses that contain a mutation in either NS5 MTase (K74I) or NS3 helicase (D290N). Thus, serotype-specific interactions either between the MTase and RdRp domains and/or between NS5 and other components of the replication complex are required for efficient viral RNA synthesis. To determine whether inter-domain interaction within NS5 plays a significant role in viral replication, we replaced the entire NS5 with DENV4 NS5 in the full-length DENV2 infectious RNA. Surprisingly, replication of the DENV2 RNA containing the DENV4 NS5 was delayed only slightly compared to the wild-type DENV2 RNA, significantly faster than replication of the chimeric RNA containing the DENV4 MTase (Fig 5A). This suggests that the serotype-specific interactions between MTase and RdRp domains are the major determinant of efficient viral replication, rather than interactions between individual NS5 domains and RNA or other proteins.

In light of the DENV NS5 structure presented here, we analyzed whether virus serotype-specific interactions exist between the MTase and RdRp domains that could explain the low viral replication of the NS5 chimera, and whether serotype-specific residues accumulate at a particular protein surface that would facilitate serotype-specific protein interactions. Multiple amino acid sequences of four DENV serotypes were analyzed to identify individual residues that are specific to each serotype. Serotype-specific NS5 residues were identified such that the residues are conserved within one DENV serotype, but not conserved among different serotypes. A total of 119 residues were identified as serotype-specific, and 38 positions out of 119 were identified for more than two serotypes (Fig 5B). These 38 residues were then mapped on the DENV3 NS5 structure (Fig 5C). Most of the residues were located on the protein surface, with several residues in the domain-domain and monomer-monomer interfaces. For example, K96, E296, and linker residues H263 and N265 are found in the domain-domain interface (Fig 5C). The more surprising discovery, however, was that many serotype-specific residues clustered on one face of the dimer, while the opposite face of the dimer, where the active sites of NS5 are exposed to solvent, contained few serotype-specific residues (Fig 5C). The best-documented example of serotype-specific differences in DENV NS5 is their nuclear localization. DENV2 and DENV3 NS5 have a functional nuclear localization signal (NLS, residues 369–406) that is recognized by importin-α/β, and predominantly localize to the nucleus [41,42]. By contrast, DENV4 NS5 does not have a functional NLS, and localizes to the cytoplasm [43]. We mapped the NLS sequence on the NS5 structure to determine whether it is located on the serotype-specific side of the dimer (Fig 5C). The NLS indeed mapped to the serotype-specific surface, and four residues within the NLS—residues 375, 383, 385, and 404—were identified as serotype-specific (Fig 5B). Thus, serotype-specific residues accumulate on a particular NS5 dimer surface that is likely mediate NS5-protein interactions that are specific for each serotype.

## Discussion

### Oligomeric state of the flavivirus NS5 in the replication complex

Genome replication in flavivirus is carried out by a membrane-bound viral replication complex that consists of viral NS proteins, viral RNA, and unknown host proteins [7]. Neither the exact composition of the replication complex nor the stoichiometry of viral NS proteins within the replication complex is currently known. The DENV NS5 structure reported here shows two dimer types with significant interface areas between the monomers (> 1,000 Å^2^). In particular, the type I dimer is formed by all eight NS5 molecules in the crystallographic ASU, even though each of these eight molecules is in a different chemical environment in the crystal (S1 Fig). The type I dimer was also observed in recently solved DENV NS5 structure [31], and thus the DENV NS5 clearly assembles into the dimer. Many NS5 residues involved in the monomer-monomer interface in the type I dimer are important for viral replication, suggesting that the NS5 dimer may be the biological unit in the replication complex. Recombinant NS5 has been shown to be a monomer in solution [35,37]. However, these experiments were performed in membrane-free, high salt (400 mM NaCl) buffers that do not necessarily reflect the cellular environment. In the membrane-bound replication complex, where free diffusion is limited to a 2-D surface, NS5 may form a higher order oligomer. Cellular interaction and pull-down assays indeed demonstrate that NS5 interacts with itself [19,38]. Furthermore, NS5 is anchored to the membrane by its interaction with NS3 in the replication complex [38]. NS3 itself does not contain any membrane-associated region, but the N-terminal protease domain requires the cofactor NS2B that forms a dimer on the membrane [38]. Thus, the membrane anchored NS5-NS3-NS2B complex could contain a dimeric form of NS5. Other viral polymerases such as poliovirus 3D, which is also a monomer in solution, oligomerize in order to facilitate efficient binding of RNA [44]. Disruption of the protein-protein interface of the poliovirus 3D oligomers led to low viral replication in infected cells, suggesting the biological significance of oligomerization [44].

### Coordination of RNA synthesis and 5’-RNA capping by flavivirus NS5

**Fig 6:**
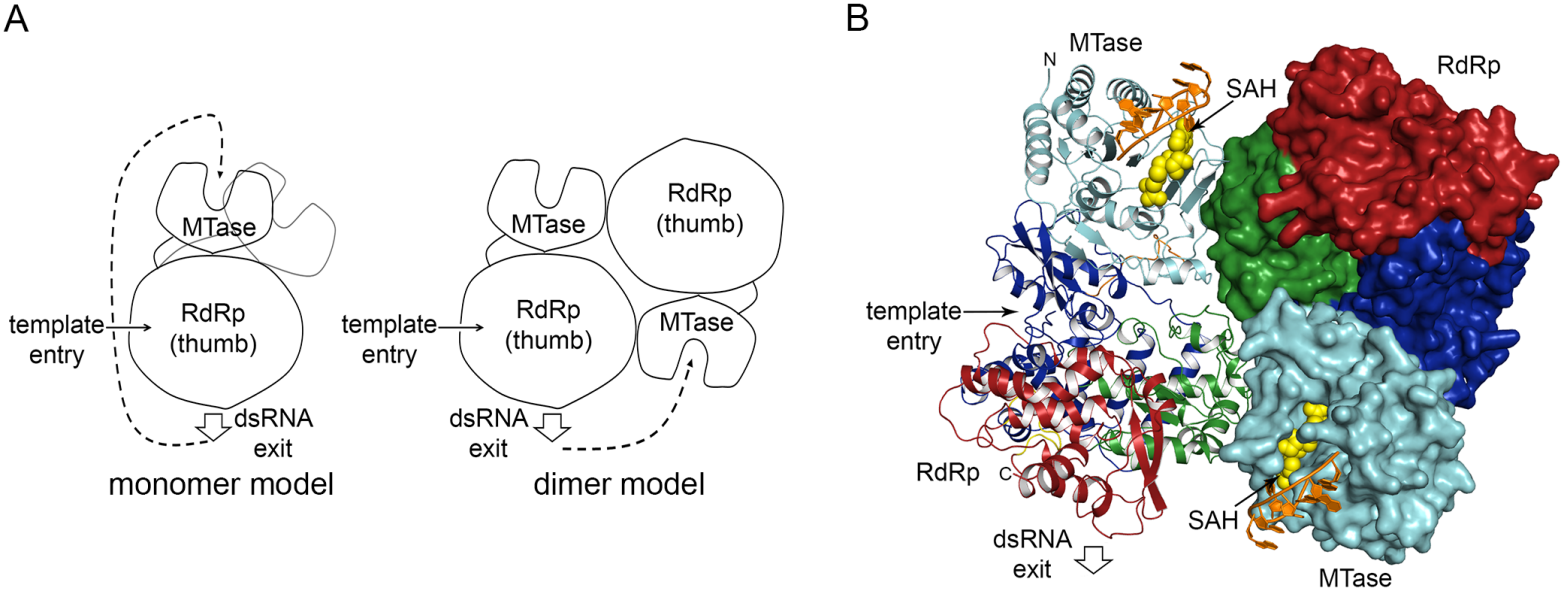
NS5 monomer and dimer models in the replication complex. **(A)** Schematics of the NS5 monomer and dimer models. Two conformations of the MTase relative to the RdRp observed in DENV and JEV NS5 structures in the monomer model are indicated by black and gray lines. The template entry and dsRNA product exit sites on the RdRp are indicated by arrows. The MTase active site is represented by a concave region. **(B)** DENV NS5 dimer with bound RNA model. To show molecular boundaries, one monomer is shown as a ribbon diagram, and the other as a molecular surface. The MTase active site is indicated by the bound SAH (yellow) and a short RNA (orange). The single-stranded RNA was modeled in by superposition of the MTase domain in our structure and the isolated MTase domain-RNA complex (PDB code 2XBM) [45].

The physical linkage of the MTase and RdRp domains in a single polypeptide of NS5 suggests that RNA synthesis and capping may be coupled during viral genome replication. In light of the dimer structure, we compared the NS5 monomer and dimer in terms of the relative orientation of the RdRp dsRNA exit and MTase active site, which could be important for the coordination of RdRp and MTase activities (Fig 6). The location of the ssRNA-binding site in the MTase domain of the full-length NS5 was identified based on superposition of the MTase domains in our structure with the structure of the isolated MTase domain-RNA complex [45] (PDB code 2XBM).

In the NS5 monomer, the dsRNA exit site of the RdRp and the active site of the MTase face opposite directions in both the DENV and JEV NS5 structures (Fig 4). Thus, the NS5 monomer model would require a large conformational change to pass the RNA from the RdRp directly to the MTase. Consequently, monomer models with multiple large conformational changes have been proposed [25,46]. Using small-angle X-ray scattering, monomeric NS5 has been shown to adopt multiple conformations in solution ranging from compact to more extended forms [35]. The DENV and JEV NS5 structures clearly indicate that NS5 adopts different arrangements of MTase and RdRp domains related by rotation along the linker (Fig 4). However, multiple large conformational changes could be limited in the replication complex, where NS5 is bound by viral and host proteins on the membrane. The NS5 dimer could thus allow coordination between MTase and RdRp domains within and across the NS5 molecules without requiring large conformational changes inherent in the monomer model. In the DENV NS5 dimer structure, the dsRNA exit site of the RdRp in one monomer and the MTase active site of its partner in the NS5 dimer face the same direction (Fig 6). The distance from the dsRNA exit of one monomer to the entrance to the MTase active site of its partner is also considerably closer than the distance to its own MTase. Thus the dsRNA product of the RdRp could easily access the MTase active site of its neighbor in the NS5 dimer (Fig 6). The dimeric form could still allow small rearrangements of MTase and RdRp domains.

How flavivirus coordinates RNA synthesis and 5’-RNA capping is not well understood. It is currently not clear how NS5 modulates multiple interactions with different RNA forms (plus, minus, double stranded, and capped RNA). In addition, following (+) strand RNA synthesis, the 5’ triphosphate end of the RNA must be dephosphorylated by NS3 5’ RNA triphosphatase activity prior to the cap addition, so NS5 is also required to interact with NS3 for viral replication. Thus, NS5 may need the flexibility to form a monomer and dimer to carry out multiple functions in the replication complex. Future effort will be directed toward validating these NS5 models. The current structure provides a framework to test how NS5 coordinates multiple reactions within a single polypeptide, and to design NS5 inhibitors of dengue virus replication.

## Materials and Methods

### Expression and purification of NS5 proteins

DENV3 NS5 constructs Δ2 and Δ6 with the priming loop shortened by either two (^797^IH^798^) or six amino acids (^795^WSIHAH^800^), respectively, were designed to make NS5 more amenable to crystallization. The plasmid encoding DENV3 NS5-Δ2 was generated from the wild-type NS5 clone (900 residues) using the QuikChange II site-directed mutagenesis kit (Agilent Technologies) with the oligonucleotide primers 5’ CCTACAAGCAGAACGACATGGTCTGCTCACCATCAGTGGATGACTAC 3’ (forward) and 5’ GTAGTCATCCACTGATGGTGAGCAGACCATGTCGTTCTGCTTGTAGG 3’ (reverse). The plasmid containing NS5-Δ2 was then used to produce NS5-Δ6 by another round of site-directed mutagenesis using the oligonucleotide primers 5’ CCCACGAGCAGAACGACACATCAGTGGATGACTACAG 3’ (forward) and 5’ CTGTAGTCATCCACTGATGTGTCGTTCTGCTCGTGGG 3’ (reverse). After DNA sequence confirmation of the NS5-Δ2 and Δ6 plasmids, the plasmids were transformed into BL21-CodonPlus-RIL *Escherichia coli* cells (Stratagene). His-tagged NS5 proteins were purified as previously reported with minor differences [35]. Briefly, 1 L of Luria Broth medium supplemented with 25 μg/ml chloramphenicol and 50 μg/ml kanamycin was inoculated with 5 mL of start culture. After reaching an OD_600_ of 0.8, the cells were induced by the addition of 1 mM isopropyl 1-thio-β-D-galactopyranoside and grown at 18°C overnight. Pelleted cells were lysed by sonication in lysis buffer [50 mM sodium phosphate pH 8.0 and 1 M NaCl, supplemented with 50 μg/mL ribonuclease A, 100 μg/mL deoxyribonuclease A, and one tablet of protease inhibitor cocktail (Roche Applied Science)]. NS5 was purified first by affinity chromatography using TALON cobalt affinity resin (Clontech) and an imidazole gradient of 5 to 150 mM in elution buffer (25 mM sodium phosphate, pH 7.0 and 500 mM NaCl). Fractions containing NS5 were concentrated to ~1 mL and further purified by size exclusion chromatography using a HiLoad 16/60 Superdex 200 preparative grade column (GE Healthcare) in 20 mM Tris-HCl pH 7.0, 300 mM NaCl, and 1 mM DTT. NS5 containing fractions were pooled. The protein concentration was determined using a Nanodrop 1000 spectrophotometer (Thermo Scientific) with a theoretical molar extinction coefficient of 217,000 M^-1^cm^-1^ and a molecular weight of 104,000 Da.

### Polymerase activity assays

Polymerase activity assays for wild-type, Δ2, and Δ6 NS5 were performed using the subgenomic RNA template as previously described [27]. Briefly, the assays were conducted in a standard reaction mixture (50 μL) containing 50 mM Tris-HCl (pH 8.0), 50 mM NaCl, 5 mM MgCl_2_, template RNA (0.2 μg; 0.2 pmol), 500 μM (each) ATP, CTP, and UTP, 10 μM unlabeled GTP, 10 μCi of [α-^32^P]GTP, and 100 μM DTT. The subgenomic RNA template (719 nt) contains both 5’- and 3’-UTR regions of the DENV2 genome. The reaction was started by adding 10 μg (100 pmol) of purified NS5 before incubation at 37°C for 1 h. The reaction was terminated by acid phenol-chloroform extraction, followed by purification on a Bio-Rad P-30 column to remove the unincorporated nucleotides. Radioactive RNA products were analyzed by formaldehyde-agarose gel electrophoresis and visualized by autoradiography. Band intensities were measured with a PhosphoImager (Molecular Dynamics).

### Crystallization and X-ray data collection

NS5-Δ2 and Δ6 proteins were concentrated to ~10 mg/mL and screened using the sitting drop vapor diffusion method in 96-well plates using a Phoenix RE liquid handling robot (Rigaku). Several conditions produced small crystals within 2 weeks for Δ2 and Δ6, which were further optimized. The best-diffracting NS5-Δ6 crystals were lens-shaped, and grew to full size within 3–7 weeks in 1.0 M succinic acid (pH 7.0), 0.1 M HEPES pH 7.0, and 1% PEG monomethylether 2000. Crystals were harvested by cryo-cooling in liquid nitrogen after soaking for ~20 seconds in well solution supplemented with 25% ethylene glycol.

X-ray diffraction data were collected at 100 K at the Advanced Photon Source beamline 21 (Argonne National Laboratory, Chicago). Four datasets were collected from three crystals. Reflections were indexed and integrated using HKL2000, and four datasets were scaled and merged together using SCALEPACK [47]. A 3.6 Å resolution cutoff was applied using CC_1/2_ and CC* values of 0.157 and 0.520, respectively, in the highest resolution shell (3.66–3.60 Å) [48]. The crystals belonged to space group *P*3_2_21 with a = b = 215.3 Å, c = 480.7 Å, and contained eight NS5 monomer in the ASU with a corresponding solvent content of 68%. The initial structure solutions were obtained by molecular replacement with DENV3 MTase (residues 7–262, PDB code 3P97) and RdRp (residues 272–883, PDB code 4HHJ) as search models using the program PHASER in the Phenix suite [49]. Seven MTase and seven RdRp solutions were found, and the eighth MTase was placed manually. After refinement with seven RdRp solutions and eight MTase, the eighth RdRp solution was found using Phenix. The eighth RdRp domain (monomer H in Fig 1) has the weakest density and is missing 133 residues. Upon initial structure solution, continuous electron densities in the *2F*_*o*_ *-F*_*c*_ map were clearly visible between the C-terminus of MTase and the N-terminus of RdRp, indicating which copies of the MTase and RdRp were part of each of eight intact polypeptide chains. The distance between the terminal ends of each corresponding MTase/RdRp pair was ~24 Å (distance between Cα atoms of R262 and N272), excluding the possibility of any other domain arrangement and making the assignment of continuous protein chains unambiguous. The *2F*_*o*_ *-F*_*c*_ map also indicated that either SAM or SAH is bound to the MTase active site. We modeled SAH in the density, because SAH was copurified in several high-resolution MTase structures [13,50], and the density for the additional methyl group of SAM was missing (S2 Fig). Additionally, the RdRp contains two metal ions coordinated by tetrahedral geometry with one metal-binding site consisting of H712, H714, C728, and C853 in the thumb subdomain, and the second site consisting of E437, H441, C446, and C449 in the fingers subdomain. Since no metal was added during crystallization, the metal ions must have come from the growth medium and been copurified with the protein. Based on the tetrahedral geometry, coordinating residues, and the positions where zinc atoms have previously been identified in the structures of other flavivirus RdRps [20,29], zinc atoms were modeled into the electron density.

Manual model building was carried out using Coot [51], and iterative cycles of refinement were carried out using phenix.refine. Initially, a global NCS was used during refinement owing to the presence of eight copies of the NS5 monomer in the ASU, which was relaxed to a torsional NCS during subsequent rounds of refinement. TLS (translation, liberation, and screw motion) refinement was also used with TLS groups automatically defined by the TLSMD server [52]. The final model contains eight full-length NS5, eight SAH, and sixteeen zinc atoms with the R and R_free_ factors of 23.8 and 27.4%, respectively (Table 1). A Ramachandran plot shows 95.4 and 4.5% of residues in allowed or generally allowed regions, and 0.1% outliers. The final model and structure factors were deposited to the Protein Data Bank with accession code 5CCV.

**Table 1:** X-ray crystallographic data collection, processing, and refinement statistics.

Data collection statistics†	
Wavelength (Å)	0.97985
No. of reflections	149,621
Space group	*P*3_2_21
Unit cell dimensions (Å)	*a = b =* 215.31, *c* = 480.68
Resolution	50.00–3.60 (3.66–3.60)*
Completeness (%)	100.0 (100.0)
Redundancy	16.4 (6.2)
*I*/*σI*	6.8 (1.0)
*R*_*p*.*i*.*m*._	0.097 (0.866)
CC_1/2_ (highest resolution shell)	0.157
CC* (highest resolution shell)	0.520
Structure refinement statistics	
Resolution range (Å)	49.2–3.60 (3.69–3.60)
*R*_*work*_ / *R*_*free*_	0.238 (0.365) /0.274 (0.399)
No. of atoms	
Protein	52,457
Ligand/ion (8 SAH/16 Zn)	224
r.m.s. deviations	
Bond lengths (Å)	0.002
Bond angles (°)	0.478
*B*-factors (Å^2^)	
Protein	97.2
Ligand/ion	97.2

*Values in parentheses are for the highest-resolution shell.

^†^Four data sets collected from three crystals were scaled and merged to produce the final statistics.

### Structure analysis

The buried surface areas and interface residues between MTase and RdRp domains and between NS5 monomers were determined using the program PISA and Contact, respectively, in the CCP4 suite [33,53]. Separate domains were delineated by the following residue ranges: MTase, residues 7–271 in DENV3 and residues 5–270 in JEV; RdRp, residues 272–892 in DENV3 and residues 274–895 in JEV. The rmsds of two structures were calculated using Pymol [54]. The conformational differences between the DENV and JEV NS5 structures were analyzed using the DynDom protein domain motion analysis program [32]. The maximum dimension of the NS5 monomer was calculated by CRYSOL [36].

### Construction of DENV2 RNA containing DENV4 NS5

Construction of a full-length cDNA of DENV2 (New Guinea C strain) and its NS5 chimera containing the DENV4 MTase in yeast/*Escherichia coli* shuttle vector were previously described [39,55]. The replacement region was amplified by PCR using primers, 5’ TCCATCATGAAGAAC ACAACCAACACGAGAAGGGGAACTGGGACCACAGGAGAG 3’ (forward) and 5’ GACCTG ACTTCTAGCCTTGTTTCATGTTAGTTTTGCCTTTTACAGAACTCCCTCACTCT 3’ (reverse), and pRS424 DENV4 cDNA (GenBank accession number M14931.2). The amplified DNA fragment was mixed with the StuI and AatII-double digested pRS424-FLDV2 cDNA encoding full-length DENV2 RNA. Yeast recombination method was used [55] to create a chimera virus cDNA having DENV4 full-length NS5 in DENV2 backbone. The chimera plasmid was linearized using the BcgI enzyme at the 3′-end of the viral sequence and was used as the template for *in vitro* transcription catalyzed by SP6 RNA polymerase (Epicenter Biotechnologies) in the presence of the 7-MeGpppG cap analog.

### Electroporation, mammalian cell culture, and immunofluorescence assay

The DENV2 RNA (∼3 μg) containing either the wild type (DENV2) NS5, a NS5 chimera (DENV4 MTase and DENV2 RdRp), or DENV4 NS5 were transfected by electroporation (Amaxa Nucleofector II system, Amaxa Biosystems, Cologne, Germany) into BHK-21 cells (American Type Culture Collection, Manassas, VA), as previously described [39]. Briefly, ∼1 × 10^6^ cells were resuspended in 100 μl of Ingenio solution (Mirus Bio, Madison, WI). After pulsing, cells were carefully transferred into prewarmed complete medium (Dulbecco's modified Eagle's medium (DMEM), supplemented with 10% fetal bovine serum and 1× streptomycin/penicillin), and allowed to recover for 5 min at 37°C in an incubator. Cells were resuspended in 10 ml of complete DMEM and incubated in a T-12.5 flask. On days 2 and 9, cells were trypsinized and transferred into a T-25 and T-75 flask, respectively. This procedure was repeated using one-third of the trypsinized cells from a T-75 flask every 5–7 days. For immunofluorescence assay, RNA-transfected cells at the end of indicated time periods were seeded into a slide (LabTek), and fixed by treatment with acetone. Cells were incubated with a 1:200 dilution of 7E11, a monoclonal antibody against DENV2 NS1. Fluorescein isothiocyante (FITC)-labeled, goat anti-mouse immunoglobulin G conjugate (Kirkegaard & Perry Laboratories) was used as a secondary antibody at a 1:100 dilution. Immunofluorescence photomicrographs (×200 magnification) were acquired using a Leitz Diaplan microscope coupled to the Leica/Wild MPS48 automated photographic system. The numbers and intensities of positive cells were compared utilizing the ImageJ program (National Institutes of Health), as previously described.

### Serotype-specific residues

Serotype-specific residues were identified in two steps. First, seven to ten NS5 sequences from each DENV serotype were randomly selected for multiple sequence alignment using Clustal W [56]. DENV1 NS5 sequences include GenBank codes AHI43715.1, ACW82945.1, ACJ04223.1, AGN94878.1, and AAK29447.1, and UniProt codes P33478.1 and P17763.2. DENV2 NS5 sequences include UniProt codes P07564.2, Q9WDA6.1, P14337.2, P12823.1, P29991.1, P14340.2, and P29990.1, and GenBank codes ABY65725.1, AII99332.1 and AHB63929.1. DENV3 NS5 sequences include GenBank codes ABV54900.1, YP_001621843.1, AHG23213.1, Q99D35.1, ACY70817.1, AAS49486.2, ABV54900.1, ABV03585.1, YP_001621843.1, and ABV54900.1. DENV4 NS5 sequences include GenBank codes GNWVDF, AHG23274.1, ACW83012.1, ACQ44391.1, ABO45246.1, AEX91754.1, and AGI95993.1. Serotype-specific residues that are conserved in each serotype, but different among serotypes were selected. Next, the virus variation resource at NCBI (http://www.ncbi.nlm.nih.gov/genome/viruses/variation/dengue/) was used to remove residues that have some variations in each serotype [57].

## Supporting Information

S1 Fig Crystal contacts of DENV NS5 dimers. Each of the four dimers within the ASU and a symmetry-generated dimer from PDB entry 4V0Q are shown in gray as a molecular surface. Crystal contacts within 4 Å of molecules from neighboring ASUs in the crystal are indicated by a colored surface.
(TIFF)

S2 Fig SAH bound in the DENV MTase active site. The *F*_*o*_-*F*_*c*_ omit map for SAH is shown as blue mesh contoured at 3σ. SAH and surrounding MTase residues are shown as yellow and cyan sticks, respectively. The RdRp domain and the linker are colored as in Fig 2A.
(TIFF)

S3 Fig Domain interface of DENV NS5. Interface residues from MTase and RdRp fingers subdomain are shown as sticks and colored as in Fig 2A. Hydrogen bonds are indicated by dashed lines.
(TIF)
