# Supplementary figures and images for: Dengue Virus Nonstructural Protein 5 (NS5) Assembles into a Dimer with a Unique Methyltransferase and Polymerase Interface
Source: PLoS Pathog. 2016 Feb 19;12(2):e1005451. doi: 10.1371/journal.ppat.1005451 (PMC4760774; doi:10.1371/journal.ppat.1005451)

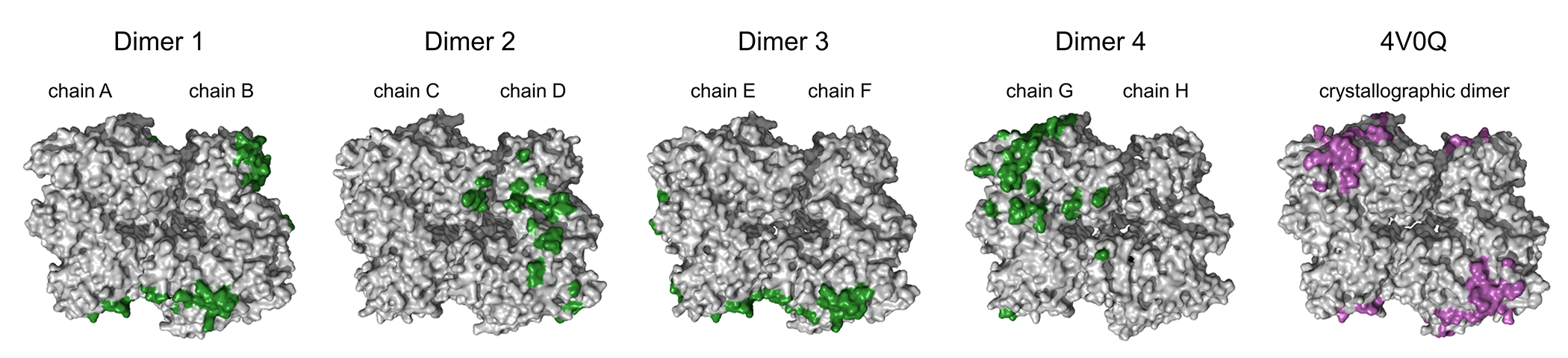

Supplement: S1 Fig — Each of the four dimers within the ASU and a symmetry-generated dimer from PDB entry 4V0Q are shown in gray as a molecular surface. Crystal contacts within 4 Å of molecules from neighboring ASUs in the crystal are indicated by a colored surface. (TIFF) [file ppat.1005451.s001.tiff]

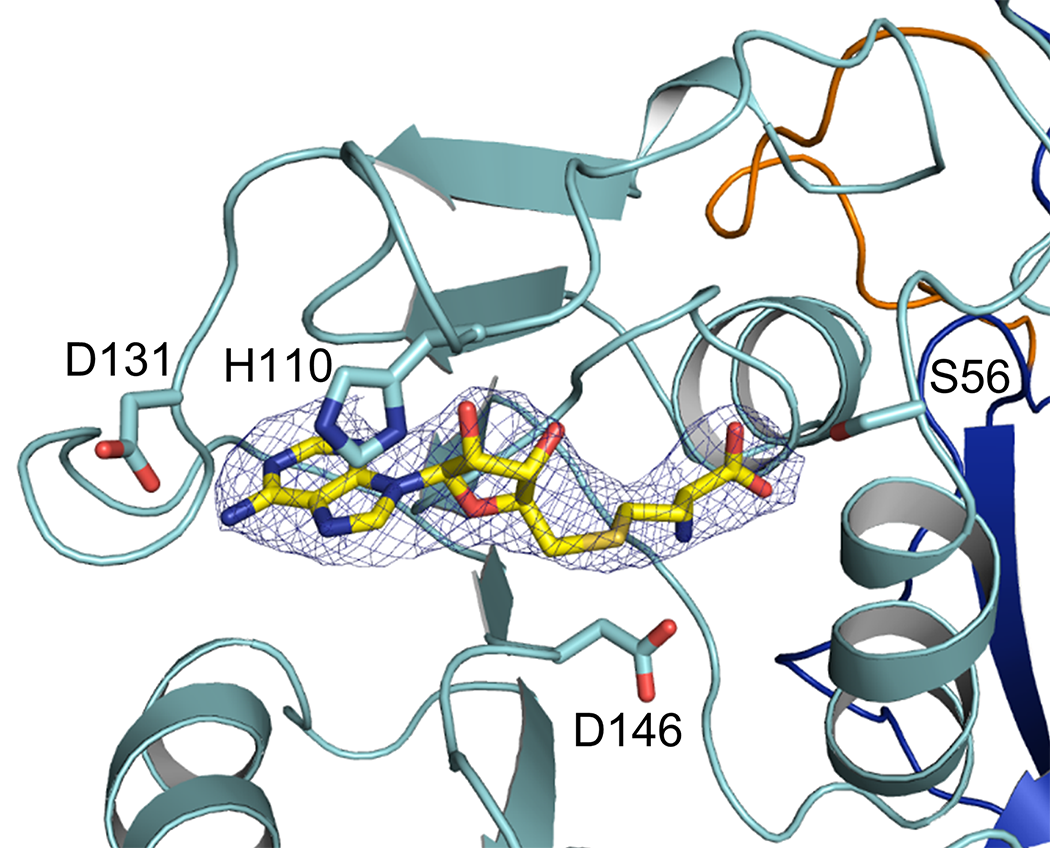

Supplement: S2 Fig — The F o-F c omit map for SAH is shown as blue mesh contoured at 3σ. SAH and surrounding MTase residues are shown as yellow and cyan sticks, respectively. The RdRp domain and the linker are colored as in Fig 2A. (TIFF) [file ppat.1005451.s002.tiff]

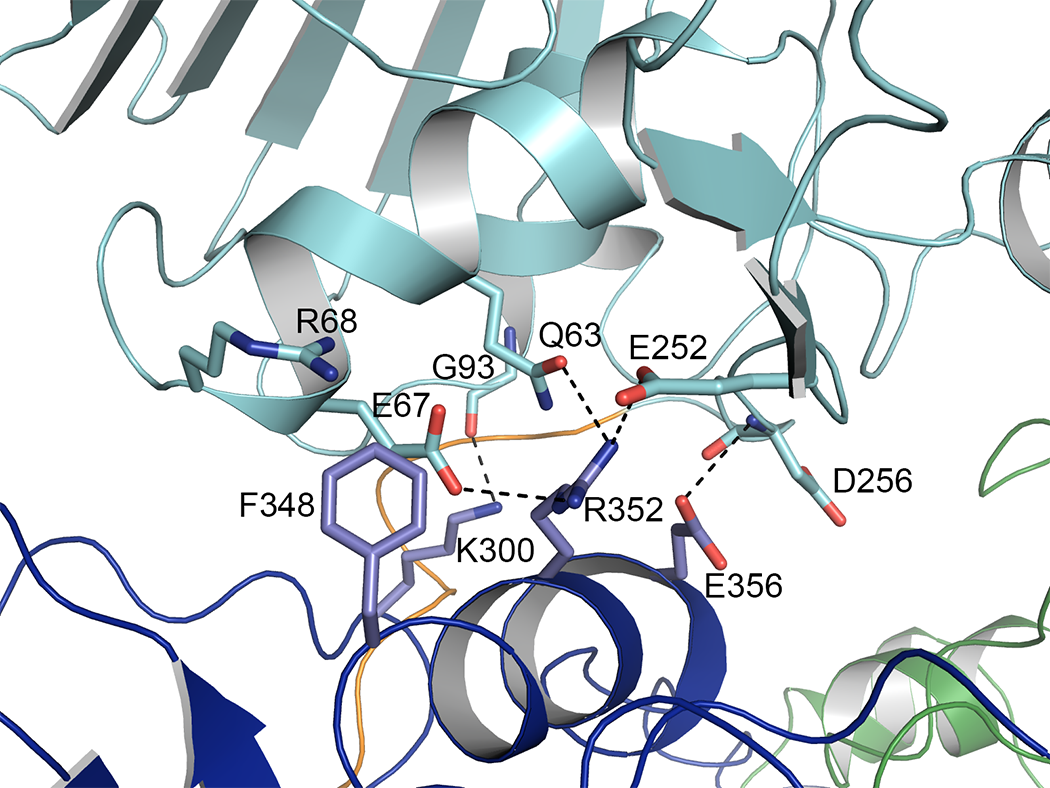

Supplement: S3 Fig — Interface residues from MTase and RdRp fingers subdomain are shown as sticks and colored as in Fig 2A. Hydrogen bonds are indicated by dashed lines. (TIF) [file ppat.1005451.s003.tif]
